# Sulcus Fixation of Foldable Intraocular Lenses Guided by Ultrasound Biomicroscopy

**DOI:** 10.1155/2015/520418

**Published:** 2015-08-30

**Authors:** Shasha Gao, Tingyu Qin, Shengnan Wang, Yong Lu

**Affiliations:** ^1^Department of Ophthalmology, The First Affiliated Hospital of Zhengzhou University, Zhengzhou 450052, China; ^2^Department of Ophthalmology, Beijing Ditan Hospital, Capital Medical University, Beijing, China

## Abstract

*Background*. To evaluate the clinical efficacy of suture fixation of foldable intraocular lens (IOL) in ciliary sulcus guided by ultrasound biomicroscopy (UBM). *Methods*. Thirty-five eyes of 32 cases needing suture fixation of foldable IOL in ciliary sulcus in our hospital were collected and divided into two groups: group A and group B. In group A, UBM was performed on 19 eyes of 17 cases before surgery to locate the projection position of ciliary sulcus in iris surface. In group B, the traditional sulcus fixation of IOL was performed on 16 eyes of 15 cases. The inserting position of needles, the haptics position of IOL and the IOL tilt, and decentration were observed by UBM examination 3 months after the surgery. Meanwhile, the vision and contrast sensitivity were analysed. *Results*. The differences in inserting position of the needle, the IOL tilt and decentration, the ratio of IOL haptics in sulcus, and uncorrected visual acuity were statistically significant (*P* < 0.05). The differences in best corrected visual acuity (BCVA) and contrast sensitivity were not statistically significant (*P* > 0.05). *Conclusions.* Sulcus fixation of foldable IOL aided by UBM can increase the accuracy of IOL haptics implanted into ciliary sulcus and reduce the IOL tilt and decentration.

## 1. Introduction

The sulcus suture fixation of the IOL was mentioned firstly by Girard [1] and Malbran et al. [2] and is widely used in the treatment of a defect of anterior or posterior capsule caused by contusion or penetration injury of the eyeball and complex surgeries. The application of a foldable IOL brings a small incision, few complications, a decrease of astigmatism, and quick recovery, significant advantages compared with a hard IOL [3]. However, because most scleral-sutured IOL procedures require that needles are placed behind the iris without direct visualization of the ciliary body, the possibility of asymmetric haptic location in the sulcus after intended sulcus implantation remains high [4].

Ultrasound biomicroscopy systems are suitable for imaging of virtually all anterior segment anatomy and pathology, including the cornea, iridocorneal angle, anterior chamber, iris, ciliary body, and lens. UBM is thus applicable for diagnostic imaging of corneal diseases, glaucoma, cysts, and tumors as well as lens implants. The ability of UBM to visualize the posterior chamber is useful for assessment of the position of the crystalline lens and lens implants which allows preoperative evaluation of the position of the sulcus before lens surgery, facilitating estimation of the postoperative intraocular lens position. In this study, we evaluated the clinical efficacy of sulcus suture fixation of the foldable IOL aided by UBM.

## 2. Patients and Methods

This study included 32 patients (35 eyes), 25 men and 7 women, who had transscleral fixation of foldable IOLs between 2012 and 2014. The mean age of the patients was 42 years with a range from 13 to 81. Eyes with infective ocular diseases, corneal diseases, severe iridocoloboma, abnormal pupil size and position, glaucoma, and retinal diseases were excluded to prevent the confounding effects in visual acuity. All patients gave their written informed consent prior to participation in the study.

The eyes were randomly divided into two groups according to the use of UBM examination. The UBM examination group (group A), including 17 cases (19 eyes), had UBM examination before sulcus fixation of foldable IOLs operation. The control group (group B), including 15 cases (16 eyes), had conventional sulcus fixation of foldable IOLs without UBM aiding. All the patients were followed up for 3 months after the surgery.

**Figure 1:**
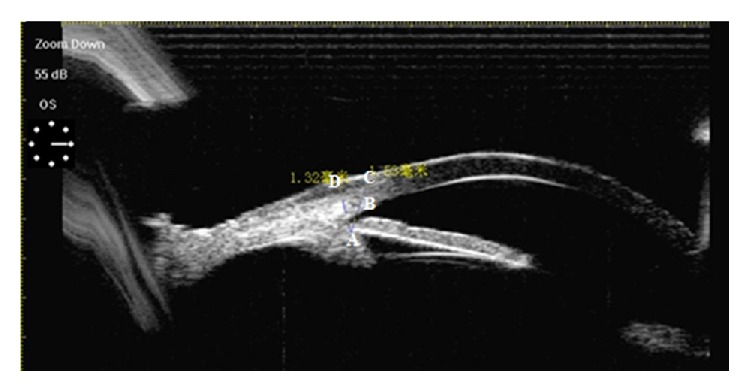
Location of sulcus by UBM before surgery (A = sulcus, B = scleral spur, C = back boundary of corneal limbus, and D = the projecting position of sulcus on the scleral surface).

The method of sulcus location was as follows: first, the full UBM picture of the position where the haptic was intended to be sutured (usually at 2 o'clock and its corresponding 8 o'clock) was reviewed. A perpendicular line was drawn from the vertex of the ciliary sulcus (the cross point between iris back surface and ciliary body) to the scleral outside surface, and the length of this line which was the distance from the ciliary sulcus to the scleral outside surface was measured. Then, the line from the sulcus vertex to the back boundary of the corneal limbus at the scleral surface was drawn and measured. Last, the distance between the projecting position of the sulcus on the scleral surface and back boundary of corneal limbus at scleral surface was calculated according to the Pythagorean theorem (Figure 1).

For surgical methods, all surgeries were performed by the same surgeon under topical anesthesia and retrobulbar anesthesia. In group A, the needle was inserted and withdrawn at the projecting position of the sulcus on the scleral surface (2 o'clock and 8 o'clock) measured by UBM and the foldable IOL was implanted and sutured traditionally in the sulcus. In group B, the inserting and withdrawing positions of the needle were 1.5 mm away from the back boundary of the corneal limbus. The other surgical steps were the same as those in group A.

**Figure 2:**
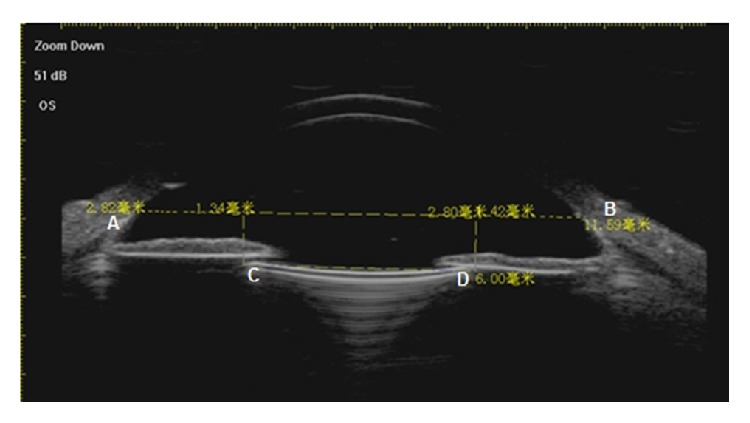
Measurement of IOL decentration (A, B = scleral spur, C, D = two optical endpoints of IOL).

**Figure 3:**
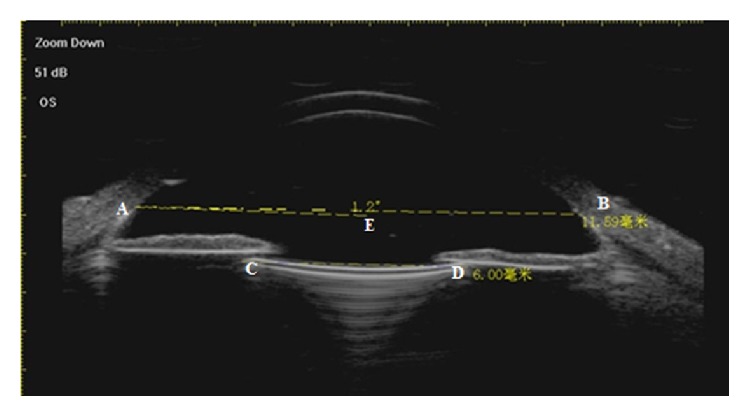
Measurement of IOL tilt (A, B = scleral spur, C, D = two optical endpoints of IOL, and line AE was the parallel line of line CD).

The UBM examination was performed on all patients 3 months after the surgery. The entire UBM image from 2 o'clock to 8 o'clock was determined. The scleral spur showed a high echo area like the olecranon in the UBM image and was the marker. The line between two olecranons of 2 o'clock and 8 o'clock was considered as the horizontal base line of the eyeball. For the IOL decentration, two perpendicular lines were drawn from both optical endpoints of the IOL to the base line and the distances between intersection points and the scleral spur were measured. The differences between these two distances were two times that of the IOL decentration (Figure 2). For the IOL tilt, a line parallel to the IOL optical endpoints connection through the left scleral spur on the UBM image was made and the angle between this line and the horizontal base line indicated the degree of IOL tilt (Figure 3).

## 3. Statistical Analysis

The Snellen preoperative and postoperative naked visual acuity (NVA) and the best corrected visual acuity (BCVA) were measured and converted into a logarithm of the minimum angle of resolution (logMAR) units for statistical analysis [5]. The contrast sensitivity at four different spatial frequencies (3, 6, 12, and 18 cycles per degree) was also measured by a CSV-1000E test lamp in a dark room and the logarithm of the result was analyzed statistically. The Wilcoxon matched pair test and Chi-square test were used for statistical analysis. A *P* value of less than 0.05 was considered statistically significant.

## 4. Results

### 4.1. The Age and Visual Acuity

The mean ages of group A and group B were 42.15 ± 22.03 and 44.27 ± 20.46 years, respectively, and the difference was not statically significant (*t* = −0.28, *P* > 0.05). The difference in preoperative and postoperative NVA and BCVA between the two groups was not statically significant. The difference in NVA between the two groups 3 months after surgery was statistically significant (Table 1).

### 4.2. The Needle Inserting and Withdrawing Position on the Scleral Surface

The needle insertion position at 2 o'clock in group A measured by UBM was 0.87 ± 0.17 mm away from the corneal limbus ranged from 0.607 mm to 1.21 mm and was different by 1.5 mm in group B (*t* = −14.49, *P* < 0.001). The needle withdrawing position at 8 o'clock in group A measured by UBM was 0.84 ± 0.18 mm away from the corneal limbus. It ranged from 0.600 mm to 1.19 mm which was different by 1.5 mm in group B (*t* = −15.26, *P* < 0.001).

### 4.3. The Haptic Position of IOL

Both haptics were in the sulcus in 9 eyes of group A (52.94%) and 2 eyes of group B. One haptic was in the sulcus in 6 eyes of group A and 6 eyes of group B. Neither of haptics was in the sulcus in 2 eyes of group A and 8 eyes of group B and the difference was statistically significant (*χ* ^2^ = 7.349, *P* = 0.025) (Table 2).

### 4.4. IOL Tilt and Decentration

The mean IOL tilt was 2.83 ± 1.10° in group A and 4.50 ± 1.78° in group B and the difference was statistically significant (*t* = −3.42, *P* = 0.002). The mean IOL decentration was 0.28 ± 0.15 mm in group A and 0.49 ± 0.20 mm in group B and the difference was statistically significant (*t* = −2.97, *P* = 0.005).

### 4.5. The Contrast Sensitivity (CS)

The sensitivity value for each spatial frequency showed no significant difference between the two groups at postoperative 3 months (Table 3).

## 5. Discussion

For correcting aphakia caused by different causes, implanting an IOL is the preferred method over glasses or contact lenses [6]. However, for the patients with inadequate capsular bag support, the transscleral sulcus fixation of the IOL is effective. Because of the blind nature of the surgical procedure, transscleral fixation also has disadvantages and blind manipulations make insertion of a needle through the ciliary sulcus more arduous.

The first practical UBM for imaging of the eye was developed by Pavlin et al. in the early 1990s [7]. UBM allows examination of the anterior segment of the eye which can show the ciliary sulcus clearly. In this study, we located the projection position of the ciliary sulcus in the iris surface by UBM and evaluated the clinical effects 3 months after the surgery. We found that the uncorrected visual acuity (UCVA) of patients in the UBM aided group was better than that in the control group, which is related to the suturing position. No statistically significant difference was found in BCVA between the two groups 3 months after the surgery. With the same measurement standard of the IOL, the difference in suturing position between two groups caused the difference of the UCVA.

For the location of the ciliary sulcus, some scholars think that 1.5 mm posterior to the back boundary of limbus is the best position of fixing the IOL [8]. But UBM examination after surgery showed that the accuracy of locating the ciliary sulcus was not high [9]. In this study, the distance between the projecting position of the sulcus on the scleral surface and the back boundary of the corneal limbus was the inserting position of the needle. The difference in inserting position between the UBM aided group and the control group was statistically significant. UBM provides the personalized positioning of the ciliary sulcus which reduces the deviation of the surgical suture caused by anatomic differences.

The UBM appearance of haptics of the IOL was similar to the sclera which was characterized by high echoes during the UBM examination. The studies before showed that the rate of haptics in the sulcus was not high. Sewelam et al. [10] performed the UBM examination on patients who underwent the sulcus fixation of the IOL and found that only 55 percent of haptics were located in the sulcus. Alp et al. [9] evaluated the efficacy of a transillumination technique for the ciliary sulcus localization in transscleral fixations of posterior chamber intraocular lenses through UBM after the surgery and the rate of haptics in the sulcus was improved to 64 percent. In our study, the rate of haptics located in the sulcus in the control group was similar to that of the previous study, but the rate in the UBM examination group was obviously higher than that in the control group and the difference was statistically significant.

The IOL decentration and tilt are current research focuses and the position of the IOL can directly affect its optical performance [11]. Oshika et al. [12] reported that major tilting of an IOL caused a substantial amount of ocular coma-like aberration which may result in skewed distortion of the object image. Asymmetric fixation of IOL is the main cause for the presence of the IOL tilt or decentration. Now purkinje and scheimpflug imaging can detect the IOL tilt and decentration [11]. In addition, Loya et al. [4] reported the using of UBM to evaluate the IOL tilt and decentration. When the optic surface of the IOL was parallel to the horizontal baseline, the IOL had no tilt and when the difference between the two distances from both optical endpoints of the IOL to the base line was over 100 *μ*m, the IOL was considered as decentration.

Contrast sensitivity measurements offer an objective measure of the quality of vision which is an important indicator for vision function [13]. The postoperative contrast sensitivity declined slightly compared with the preoperative one but there was no statistically significant difference between the two groups. The explanation might be due to the skilled operator. In both groups, the average IOL decentration and tilt were less than 0.5 mm and 5°, respectively, which caused little impact on the vision quality [14].

## 6. Conclusion 

UBM guided sulcus fixation of foldable IOL increases the accuracy rate of haptics located in the sulcus and decreases the IOL decentration and tilt. The current study was limited by the short follow-up and small cases and the long term effect needs further investigation.

## Figures and Tables

**Table 1:** Comparison of VA between two groups (logMAR).

	Preoperation		Postoperation	
	NVA	BCVA	NVA	BCVA
Group A	1.15 ± 0.25	0.27 ± 0.12	0.23 ± 0.11	0.14 ± 0.06
Group B	1.15 ± 0.27	0.29 ± 0.15	0.34 ± 0.15	0.15 ± 0.05
*P* value	0.81	0.78	0.03	0.7

VA = visual acuity, NVA = naked visual acuity.

BCVA = best corrected visual acuity.

**Table 2:** Haptic location 3 months after surgery.

	Both haptics in sulcus	One haptic in sulcus	No haptic in sulcus	*P* value
Group A	9	6	2	0.025
Group B	2	6	8	

**Table 3:** Contrast sensitivity 3 months after surgery.

	3 c/d	6 c/d	12 c/d	18 c/d
Group A	1.66 ± 0.14	1.73 ± 0.15	1.39 ± 0.20	0.74 ± 0.17
Group B	1.65 ± 0.15	1.72 ± 0.15	1.38 ± 0.19	0.73 ± 0.17
*P* value	0.47	0.94	0.81	0.83
